# Cardiovascular autonomic alterations in hospitalized patients with community-acquired pneumonia

**DOI:** 10.1186/s12931-016-0414-8

**Published:** 2016-08-04

**Authors:** Stefano Aliberti, Eleonora Tobaldini, Fabio Giuliani, Vanessa Nunziata, Giovanni Casazza, Giulia Suigo, Alice D’Adda, Giulia Bonaiti, Andrea Roveda, Andreia Queiroz, Valter Monzani, Alberto Pesci, Francesco Blasi, Nicola Montano

**Affiliations:** 1 Department of Pathophysiology and Transplantation, University of Milan, Fondazione IRCCS Ca’ Granda, Ospedale Maggiore Policlinico, Via F. Sforza 35, Milan, Italy; 2 Departments of Internal Medicine, and Clinical Sciences and Community Health, University of Milan, Fondazione IRCCS Ca’ Granda, Ospedale Maggiore Policlinico, Via F. Sforza 35, Milan, Italy; 3 Department of Biomedical and Clinical Sciences “L. Sacco”, University of Milan, Via Giovanni Battista Grassi 74, Milan, Italy; 4 Health Science Department, University of Milan Bicocca, Clinica Pneumologica, AO San Gerardo, Via Pergolesi 33, Monza, Italy; 5 Department of Physical Education, Federal University of Juiz de Fora, Minas Gerais, Brazil; 6 Department of Emergency Medicine, Fondazione IRCCS Ca’ Granda, Ospedale Maggiore Policlinico, Via F. Sforza 35, Milan, Italy

**Keywords:** Pneumonia, Cardiac autonomic control, Sympathetic, Heart rate variability, Spectral analysis, Symbolic analysis

## Abstract

**Background:**

Alterations of cardiac autonomic control (CAC) are associated with poor outcomes in patients with infectious and non-infectious diseases. No evaluation of CAC in patients with community-acquired pneumonia (CAP) has been performed so far. The aim of the study was to assess CAC in patients with CAP and evaluate the impact of its alterations on disease severity and clinical outcomes in a multicenter, prospective, observational study.

**Methods:**

Consecutive patients hospitalized for CAP were enrolled between 2011 and 2013 two university hospitals in Italy. CAC was assessed by linear spectral and non-linear symbolic analysis of heart rate variability. The presence of severe CAP was evaluated on hospital admission. The primary study outcome was time to clinical stability (TCS) during hospitalization.

**Results:**

Among the 75 patients enrolled (median age: 75 years; 57 % males), a significantly lower total variability and reduction of sympathetic rhythmical component with predominant respiratory modulation was detected in comparison to controls. Among CAP patients affected by a severe CAP on admission, CAC showed a lower sympathetic modulation and predominant parasympathetic oscillatory rhythm. At the multivariate analysis, variables independently correlated with a TCS >7 days were total power, as marker of total variability, [OR (95 % CI): 0.997 (0.994–1.000), *p* = 0.0454] and sympathetic modulation [OR (95 % CI): 0.964 (0.932–0.998), *p* = 0.0367].

**Conclusions:**

Loss of sympathetic rhythmical oscillation is associated with a more severe disease and worse early clinical outcome in hospitalized patients with CAP.

**Electronic supplementary material:**

The online version of this article (doi:10.1186/s12931-016-0414-8) contains supplementary material, which is available to authorized users.

## Background

Community-acquired pneumonia (CAP) represents the first cause of death for infectious diseases in western countries, with a mortality rate that has remained stable since the introduction of antibiotics in the 1940s [1]. During the past years, several efforts have been focused on exploring new approaches and testing strategies to reduce mortality by reducing complications related to pneumonia. Recent evidence has particularly shown that cardiovascular events (CVE) might occur in up to 30 % of hospitalized patients with CAP leading to an increase short and long-term mortality [2–5]. A better understanding of the physiopathological pathways sustaining the association between CAP and CVE is still needed in order to target interventions to improve patients’ outcomes.

It has been hypothesized that an alteration of cardiac autonomic control (CAC), as assessed by heart rate variability (HRV), may play a key role among the complex interaction of events that may lead to the occurrence of CVE during CAP [4–8]. This alteration is characterized by a reduction of total variability and an altered sympatho-vagal balance. Previous literature reported an alteration of CAC in several cardiovascular diseases, including congestive heart failure and myocardial infarction, and it has shown that it is strongly associated with poor outcomes [9–11]. The impact of infections on CAC has been evaluated so far only in patients with severe sepsis who show a lower total HRV and impaired sympathetic modulation [12]. No previous experiences have been designed to study CAC in a specific population of patients with CAP.

The aim of this study was to evaluate CAC in hospitalized patients with CAP and particularly the impact of possible autonomic alterations on both disease severity on admission and early clinical outcomes.

## Methods

### Study design and population

This was a pilot, multicenter, prospective, observational study on consecutive patients who were hospitalized because of an episode of CAP at the Respiratory Units of the Policlinico Hospital in Milan and the San Gerardo Hospital in Monza, Italy, from September 2011 to January 2013. The study was approved by the San Gerardo Hospital institutional review boards (IRB) in Monza (FAILCAP;10.2.2011) and the IRCCS Fondazione Cà Granda Policlinico IRB (nr.1686;FAILCAP) in Milan. An informed consent was signed by all participants. Patients with a diagnosis of CAP who showed a stable sinus rhythm on the electrocardiogram (ECG) performed on hospital admission were included in the study. Patients with a diagnosis of healthcare-associated pneumonia were included in the study [13]. Patients with at least one among the following were excluded from the study: 1) pneumonia that developed in a patient who was discharged from the hospital within the prior 14 days of hospital admission; 2) absence of sinus rhythm on ECG at hospital admission; 3) patients with pacemaker rhythm on ECG at hospital admission; 5) patients undergoing mechanical ventilation, in whom the application of a positive pressure might alter the evaluation of CAC; 6) patients on chronic treatment with inhaled long acting either muscarinic agents (LAMA) or beta agonists. Subjects without chronic respiratory, cardiovascular, and metabolic diseases matched for age and sex with the CAP group were recruited in a general practitioner clinic during the same period and enrolled as Control group. These subjects were on neither respiratory nor cardiovascular medications.

### Data collection

The clinical management of patients, laboratory evaluations and antimicrobial therapy were performed according to the current standard of care for patients with CAP [14]. Patients were seen daily during their hospital stay by one or more of the investigators who recorded clinical data, see Additional file 1. Microbiological examinations were performed on sputum, urine, and blood during the first 24 h after admission and according to standard of practice, see Additional file 1 [15]. Empiric antibiotic therapy was administered in compliance with the European Respiratory Society guidelines [14]. Severe CAP was defined according to the 2007 American Thoracic Society/Infectious Diseases Society of America guidelines [16].

### Assessment of cardiac autonomic control

In order to evaluate the cardiac autonomic control, patients underwent the recording of ECG and respiratory movements using a thoracic piezoelectric belt before empiric antibiotic treatment and within the 6 h after admission to the hospital. Control group underwent the recording of ECG and respiratory movements using the same device (see Additional file 1).

Cardiac autonomic control was evaluated using a classical linear spectral analysis and a more recent non-linear symbolic analysis [6]. Spectral analysis assesses the rhythmic oscillatory components that characterize the heart period time series and it identifies three main oscillations embedded in heart period time series: very low frequency component (VLF), low frequency component (LF) marker of sympathetic modulation and high frequency component (HF), synchronous with respiration and marker of vagal modulation. On the other hand, symbolic analysis is a non-linear method able to evaluate autonomic cardiac modulation and to detect non-reciprocal changes of sympathetic and parasympathetic modulations on heart period time series [17–20]. Mathematical and technical details are reported in the Additional file 1.

### Study outcomes

In order to evaluate the impact of alterations of CAC on pneumonia-related outcomes, time to clinical stability (CS) has been identified as the primary study outcome, see Additional file 1 [21, 22]. Secondary study outcomes were cardiovascular events (CVE), length of hospital stay (LOS) and 30-day mortality. Types and definitions of CVE are reported in the Additional file 1. LOS was calculated as number of days from the date of admission to the date of discharge. Thirty-day mortality was considered if death by any cause occurred during the first 30 days after the diagnosis of pneumonia. Mortality for patients who have been discharged before 30 days after the diagnosis of pneumonia was evaluated by a phone call at 30 days.

### Statistical analysis

Descriptive statistics were reported at baseline, with continuous data expressed as means ± standard deviation for normally distributed data or as median (interquartile range -IQR) for skewed data, and categorical data expressed as counts. Patient characteristics were compared between groups: patients with severe CAP versus patients with non-severe CAP; patients with TCS ≤7 days versus patients with TCS >7 days. Differences of continuous data between groups were evaluated by unpaired *t*-test or Mann–Whitney *U* test. Univariate and multivariate logistic regression analyses were performed to assess association between the time to clinical stability (dichotomized, ≤7 vs. >7 days) and the following variables: heart rate (HR), total power (TP), VLF, LFa, HFa, LFnu, HFnu, LF/HF, central frequency of respiration (RESPHF), 0V%, 1V%, 2LV%, 2UV% and Pneumonia Severity Index (PSI), see the Additional file 1 [22, 23]. Sample size calculation is also reported in the Additional file 1. A p value <0.05, two sided, was considered statistically significant. All the statistical analyses were performed with SAS statistical software (release 9.4; SAS Institute Inc, Cary, NC).

## Results

### Study population

Among the 105 patients who were screened during the study period, 30 were excluded: 10 refused to give the informed consent, 6 had cardiac arrhythmias on hospital admission, 6 underwent mechanical ventilation on admission, 4 had a pacemaker and 4 were on LAMA. The final study population was composed by 75 patients (median age: 75 years; 57 % males). Baseline demographics, comorbidities, disease severity, clinical and laboratory findings on admission, microbiology, and antibiotic therapy of the study population are summarized in Table 1. A total of 26 healthy controls (median age: 68 years; 58 % males) were enrolled during the same period.

**Table 1:** Baseline demographics, comorbidities, disease severity, clinical and laboratory findings on admission, microbiology, and antibiotic therapy of the study population

	Study population *n* = 75
Demographics, *n*. (%)	
Male	43 (57)
Age, median (IQR) years	75 (59–84)
Body mass index, median (IQR)	24 (20–27)
Healthcare-associated pneumonia	5 (7)
Comorbidities, *n*. (%)	
Active neoplastic disease	12 (16)
Chronic obstructive pulmonary disease	18 (24)
Diabetes mellitus	11 (15)
Cerebrovascular accident	9 (12)
Liver disease	5 (7)
Neurological diseases	11 (15)
Renal disease	8 (11)
Chronic renal failure	6 (8)
Family history of coronary artery disease	16 (21)
Essential arterial hypertension	36 (48)
Congestive heart failure	7 (9)
Active coronary artery disease	12 (16)
Prior acute myocardial infarction	10 (13)
Atrial fibrillation	2 (3)
Hyperlipidemia	14 (19)
Medications before admission, *n*. (%)	
Aspirin	17 (23)
Beta-blockers	13 (17)
Angiotensin-converting-enzyme inhibitors	14 (19)
Antiplatelets	8 (11)
Statins	12 (16)
Severity on admission, *n*. (%)	
Mental status change	4 (5)
PSI Risk Class IV and V	54 (72)
PSI Risk Class V	21 (28)
Acute respiratory failure	39 (52)
Severe sepsis	14 (19)
Clinical and laboratory data on admission, median (IQR)	
Heart rate, bpm	80 (71–92)
Respiratory rate, bpm	20 (18–25)
Systolic blood pressure, mmHg	128 (115–146)
Diastolic blood pressure, mmHg	70 (60–80)
White blood cells, cell/L^−1^	11,840 (9180–15,900)
Hemoglobin, g/dL	12.6 (11–14.2)
Hematocrit, %	37 (33–41)
Albumin, g/dL	3.3 (3.1–3.8)
Platelets, cell/L^−1^	196,000 (161,000–289,000)
Lactate dehydrogenase, mg/dL	378 (318–444)
Blood urea nitrogen, mg/dL	40 (27–49)
Creatinine, mg/dL	1 (0.8–1.3)
Sodium, mEq/L	136 (133–139)
Potassium, mEq/L	4 (3.7–4.4)
Glucose, mg/dL	119 (106–146)
C-reactive protein, mg/L	12.9 (6.25–28.8)
pH	7.46 (7.44–7.48)
Microbiology, *n*. (%)	
Isolated pathogen	13 (17)
*S. pneumoniae*	8 (11)
*S. aureus*	2 (3)
*Legionella pneumophila*	2 (3)
*P. aeruginosa*	1 (1)
Empiric antibiotic treatment, *n*. (%)	
Ceftriaxone	36 (48)
Azithromycin	38 (51)
Levofloxacin	32 (43)
Ceftazidime	8 (11)
Piperacillin/tazobactam	9 (12)
Others	23 (30)

*n.* number, *IQR* 25–75 interquartile range, *PSI* pneumonia severity index

### Cardiac autonomic control in CAP patients

In comparison to controls, CAP patients were characterized by significantly higher HR and by a significant reduction of total variability, as shown by lower level of total power and VLF component. As to sympatho-vagal balance, CAP patients showed lower level of LFnu, marker of sympathetic modulation, and an increase of 2UV%, marker of parasympathetic modulation, in comparison to controls, see Table 2.

**Table 2:** Autonomic parameters evaluated by both spectral and symbolic analysis in patients with community-acquired pneumonia (CAP) and controls

	CAP patients *n* = 75	Controls *n* = 26	*p*
Parameter			
Heart rate, median (IQR) bpm	80 (71–92)	68 (60–73)	0.006
Spectral analysis			
Total power, median (IQR) ms^2^	159 (79–368)	522 (335–1760)	<0.001
VLF, median (IQR) ms^2^	49 (0–121)	262 (99–983)	<0.001
LFnu, median (IQR)	32 (11–63)	56 (37–75)	0.008
HFnu, median (IQR)	42 (13–59)	41 (22–55)	0.935
LF/HF, median (IQR)	0.92 (0.23–2.6)	1.3 (0.73–3.1)	0.120
HF Hz, median (IQR)	0.31 (0.27–0.37)	0.29 (0.26–0.33)	0.096
Symbolic analysis			
0V, median (IQR) %	26 (13–38)	30 (12–46)	0.451
1V, median (IQR) %	39 (33–47)	44 (38–49)	0.077
2LV, median (IQR) %	4.4 (2.2–8.5)	6.0 (3.5–12)	0.164
2UV, median (IQR) %	24 (12–41)	17 (12–24)	0.020

*n.* number, *IQR* 25*–*75 interquartile range, *bpm* beats per minute, *ms* ^*2*^ milliseconds^2^, *VLF* very low frequency, *LF* low frequency, *HF* high frequency, *nu* normalized units, *Hz* Hertz

### Cardiac autonomic control and CAP severity on admission

**Fig. 1:**
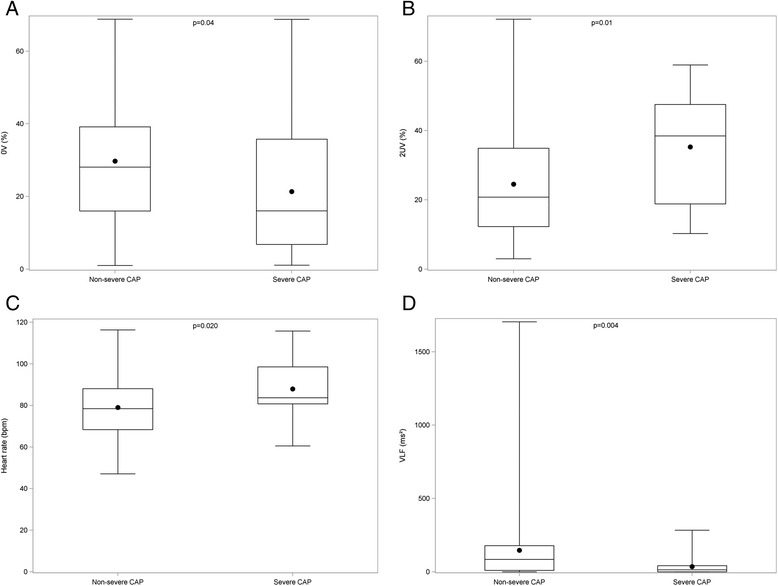
Evaluation of cardiac autonomic control in the study population, according to presence of severe community-acquired pneumonia (CAP)

A total of 19 patients (25 %) had severe CAP on hospital admission. Complete data on cardiac autonomic modulation of the study population according to different severity of the disease on hospital admission are reported in Additional file 1: Table S1. In comparison to patients without severe CAP, those with severe CAP on admission showed a significant higher median HR [78 (68–88) vs. 83 (80–98) bpm, *p* = 0.021], significant lower median VLF component [84 (9–177) vs. 13 (0.1–41) ms^2^, *p* = 0.004], significant median lower 0V% [28 (16–39) vs. 16 (6–35), *p* = 0.047], marker of sympathetic modulation, and significant higher median 2UV% [20 (12–35) vs. 38 (19–47), *p* = 0.011], marker of parasympathetic modulation, suggesting a global shift of the sympatho-vagal balance towards vagal predominance (see Fig. 1). The univariate logistic regression analysis for the presence of severe CAP on hospital admission is reported in Additional file 1: Table S2. At the multivariate analysis, variables independently correlated to severe CAP on admission were HR [OR (95 % CI): 1.078 (1.025–1.134), *p* = 0.004] and 2UV% [OR (95 % CI): 1.042 (1.001–1.084), *p* = 0.044], c-statistic for the multivariate model: 0.804.

As additional analysis, we compared autonomic control in CAP patients with vs. without severe sepsis. As shown in Table 1, we had in our sample 14 patients (19 %) with severe sepsis and 61 (81 %) without severe sepsis. The comparison of autonomic parameters between these two groups showed that heart rate was higher in the severe sepsis group compared to non severe sepsis group (88 vs 79 bpm, *p* = 0.04) and only 2UV%, symbolic marker of parasympathetic modulation, was higher in the severe sepsis group compared to non severe sepsis group (36 vs 25 %, *p* = 0.01).

### Cardiac autonomic control and CAP outcomes

A total of 48 patients (64 %) reached clinical stability before 7 days of hospital admission. In comparison to patients who reached CS within 7 days, those who reached CS after 7 days had significant lower total power (*p* = 0.001) and lower VLF component (*p* = 0.003). A complete report of both spectral and symbolic analysis is depicted in Table 3. The univariate logistic regression analysis for TCS >7 days is reported in Additional file 1: Table S3. At the multivariate analysis, variables independently correlated with a TCS >7 days were total power [OR (95 % CI): 0.997 (0.994–1.000), *p* = 0.045], 0V% [OR (95 % CI): 0.964 (0.932–0.998), *p* = 0.037] and Resp HF [OR (95 % CI): 1.092 (1.022–1.167), *p* = 0.009], c-statistic for the multivariate model: 0.813.

**Table 3:** Spectral and symbolic analysis among patients who reached clinical stability within 7 days vs. after 7 days from hospital admission

	TCS ≤7 days *n* = 48	TCS >7 days *n* = 27	*p*	p*
Parameters, *n*. (%)				
Heart rate, median (IQR) bpm	79 (70–89)	81 (72–96)	0.305	0.712
Spectral analysis				
Total power, median (IQR) ms^2^	233 (105–433)	94 (59–214)	0.002	0.001
VLF, median (IQR) ms^2^	87 (11–211)	20 (0–66)	0.001	0.003
LFnu, median (IQR)	32 (15–63)	30 (4.3–68)	0.611	0.823
HFnu, median (IQR)	43 (14–57)	36 (12–63)	0.795	0.823
LF/HF, median (IQR)	0.97 (0.25–2.4)	0.78 (0.06–6.9)	0.497	0.618
HF Hz, median (IQR)	0.30 (0.27–0.34)	0.34 (0.29–0.42)	0.041	0.106
Symbolic analysis				
0V, median (IQR) %	28 (16–48)	18 (10–30)	0.026	0.059
1V, median (IQR) %	41 (34–46)	37 (31–49)	0.732	0.674
2LV, median (IQR) %	3.7 (1.7–6.7)	6.7 (3.6–11.7)	0.041	0.149
2UV, median (IQR) %	20 (12–35)	32 (18–45)	0.017	0.092

* after adjustment for the presence vs. absence of severe CAP on hospital admission

*n.* number, *IQR* 25–75 interquartile range, *TCS* time to clinical stability, *bpm* beats per minute, *ms* ^*2*^ milliseconds^2^, *VLF* very low frequency, *LF* low frequency, *HF* high frequency, *nu* normalized units, *Hz* Hertz

Among the study population one patient experienced a myocardial infarction, two patients an acute cardiogenic pulmonary edema and two patients a new cardiac arrhythmia during hospitalization. All these patients experiencing CVE reached CS after 7 days of hospitalization. The median (IQR) LOS in the entire study population was 9 (7–14) days. A total of 5 patients died within 30 days after the diagnosis of pneumonia.

## Discussion

This pilot study demonstrates that cardiac autonomic profile of CAP patients is characterized by a lower total variability and loss of sympathetic rhythmical component with predominant respiratory modulation in comparison to controls. Among patients with severe CAP, cardiac autonomic control shows a lower sympathetic modulation and predominant parasympathetic oscillatory rhythm. Finally, after adjustment for the severity of the disease on admission, CAP patients showing a delay in reaching clinical stability, had lower total power and lower sympathetic modulation on hospital admission.

Our data seem to be in line with previous experiences performed in septic patients. During sepsis, a lower total variability, which represents the capability of the cardiovascular system of responding to external perturbations and impaired sympathetic modulation characterizes CAC and the lower the total variability, the more severe is sepsis [12, 24]. So far, no data have been reported on the evaluation of the cardiac autonomic control in a homogenous population of hospitalized patients with pneumonia. A common expectation would be an increase of sympathetic activity, as previously hypothesized [8]. However, we found a loss of rhythmical properties of sympathetic discharge and a predominant respiratory modulation in CAP patients. These finding suggest that the events that occur during pneumonia, including a systemic inflammatory response and impairment in gas exchange, could affect the central rhythmic organization of autonomic control. This is similar to what occurs in patients with congestive heart failure (CHF). Studies in patients with CHF evaluating muscle sympathetic nerve activity (MSNA) showed a high sympathetic activity, positively correlated with the severity of the disease, but a lower total variability and sympathetic oscillation, with a relative predominance of the parasympathetic oscillation [25]. In CHF patients, this occurs when central and peripheral reflexes, mediated by respiration, baroreceptors and chemoreceptors, loose their ability to rhythmically inhibits central sympathetic activity. Therefore, a parallel decrease in LFnu modulation and total variability is a marker of an increased sympathetic activity associated with the loss of its physiological rhythmical properties, and this is associated with a poorer prognosis [11, 19, 25].

Inflammatory reflex, through the activation of *vagus* nerve, controls the immune response to injury [26]. Alterations of CAC we found in CAP patients could be seen as the final consequence of a complex interaction between systemic inflammation, that could trigger the inflammatory reflex, and hypoxia, that could trigger central and peripheral autonomic reflexes. Autonomic alterations in this population, characterized by a lower level of total variability and a reduction of LF components, could be partially related to the activation of the above mentioned excitatory different reflexes and these results were more evident in patients with severe CAP, compared to those without severe CAP on admission. In summary, CAP patients with a more severe disease on admission seem to show a loss of rhythmic sympathetic oscillation, with a predominant respiratory oscillation characterizing HRV. These findings were consistent across different evaluations of the severity of the disease on admission.

Finally, when we evaluated an early clinical outcome in our population, we found that total power and 0V% were significantly lower among those with a delay in reaching clinical stability, as confirmed in the multivariable approach. We already know from previous literature that a reduction of total power is strongly associated with poor outcomes in several cardiovascular diseases, including myocardial infarction, CHF, and major arrhythmias [10, 11]. Few other studies identified HRV as prognostic factor in patients with sepsis. A reduction of total variability and LF and higher HF are associated with poor in-hospital outcome and poor short-term prognosis in septic patients [12, 24, 27, 28].

Our results might have important clinical implications in light of the occurrence of cardiovascular events in up to 30 % of hospitalized patients with CAP with an increase in both short and long-term mortality [2]. Previous literature has showed that a deregulation of the sympatho-vagal balance increases cardiovascular risk in cardiac patients [9–11]. Our results provide important physiopathological elements, which can be used for the risk stratification of CAP patients. We could speculate that a deregulation of CAC characterized by a reduction of total variability and loss of sympathetic rhythmical property could be related and/or predict the onset of cardiovascular events and mortality in CAP patients. Now, we cannot speculate that a specific treatment directed towards alteration of CAC might help in reducing cardiovascular complications in CAP patients. We suggest future ad hoc clinical studies to correlate the autonomic dysfunction to cardiovascular morbidity and mortality in CAP patients as well as interventional studies to restore sympathetic rhythmical discharge are required.

One of the major limitations of our study was the absence of a direct evaluation of the sympathetic activity using MSNA. However, MSNA is an invasive technique that is difficult to apply in acute patients. We decided to evaluate CAC only on admission, while other evaluations during hospitalization or even after hospital discharge could provide information during both treatment and recovery from pneumonia. Biochemical assessments of serum cytokines levels or other markers of systemic inflammation have not been performed. The analysis and interpretation of HRV in CAP patients may be challenging because when breathing is more vigorous, respiratory fluctuations may dominate HRV. Paced breathing could have added additional information in our study. We included CAP patients independently from the presence of cardiovascular comorbidity in order to evaluate a population close to real life. Among them, some were on beta-blockers that might affect CAC. Due to small sample size, we were not able to correct for this important confounder, while we suggest future studies including a larger sample of CAP patients to take into account not only beta-blockers but also other drugs, which could possibly affect CAC. In addition, a sub-group analysis on patients with bacteremia would have been very important, but unfortunately, we had only 3 patients with bacteremia, and we were not able to perform any specific analysis on them. Finally, the small sample size allowed us to investigate neither a specific population of bacteraemic patients nor a possible relationship between CAC and pathogens causing pneumonia.

On the other hand, this was the first pilot experience evaluating the cardiovascular autonomic modulation in hospitalized patients with CAP according to both different markers of the severity of the diseases and early clinical outcome. All the findings were consistent through the evaluation of the severity of the disease on admission and clinical outcomes and our results were strengthened by the use of two different tools, spectral and symbolic analysis, able to provide complementary information on CAC.

## Conclusions

CAC is altered in CAP patients, with a lower sympathetic modulation and a predominant parasympathetic oscillatory rhythm, especially among those with severe CAP. CAP patients showing a delay in reaching clinical stability during hospitalization show a significant lower total variability on hospital admission. Our results may provide important physiopathological elements to stratify CAP patients in terms of disease severity and cardiovascular morbidity and mortality. Further studies should be focused on the evaluation of the impact of CAC on the occurrence of cardiovascular events both during hospitalization and after discharge and on late clinical outcomes in larger cohorts of CAP patients.

## Additional file

Additional file 1: Autonomic cardiac parameters in patients with and without severe community-acquired pneumonia (CAP) on hospital admission. (DOC 84 kb)

## Abbreviations

CAC: cardiac autonomic control
CAP: community-acquired pneumonia
CHF: congestive heart failure
CS: clinical stability
CVE: cardiovascular events
ECG: electrocardiogram
HF: high frequency
HR: heart rate
HRV: heart rate variability
IQR: interquartile range
LAMA: long acting muscarinic agents
LF: low frequency
LOS: length of hospital stay
MSNA: muscle sympathetic nerve activity
OR: odds ratio
PSI: Pneumonia Severity Index
RESPHF: central frequency of respiration
TCS: time to clinical stability
TP: total power
VLF: very low frequency component
