# Supplementary material for: Cardiovascular autonomic alterations in hospitalized patients with community-acquired pneumonia
Source: Respir Res. 2016 Aug 4;17:98. doi: 10.1186/s12931-016-0414-8 (PMC4973093; doi:10.1186/s12931-016-0414-8)
Supplement: Additional file 1: — Autonomic cardiac parameters in patients with and without severe community-acquired pneumonia (CAP) on hospital admission. (DOC 84 kb) [file 12931_2016_414_MOESM1_ESM.doc]

**Additional file 1**

**Cardiovascular autonomic alterations in hospitalized patients with community-acquired pneumonia**

**MATERIALS AND METHODS**

**Data collection**

Charts of all patients were abstracted using a standardized form. Demographic characteristics, comorbidities, clinical, and laboratory results on admission and during hospitalization and severity of the disease on admission including the pneumonia severity index.^1^

**Evaluation of the cardiac autonomic control**

Cardiorespiratory variables were recorded for ten minutes in supine position. In this study, we used short segments of 5 minutes length for spectral and symbolic analysis. Several studies evaluated the reliability of short-term heart rate variability (HRV) analysis and concluded that short-term HRV can be considered a reliable tool to investigate autonomic cardiovascular control.^2^ Both patients and controls were freely breathing but they were not allowed to talk during the signal acquisition. Electrocardiogram (ECG) and respiration were recorded using a telemetric system device (BT16, Ditta Marazza, Monza, Italy). Using a Bluetooth technology, this system transfers the recorded signals to a computer using a conversion card A/D, with *ad hoc* acquisition program. The ECG was acquired using a 1000 Hz sample frequency.

To obtain time series of consecutive RR intervals to which apply heart rate variability (HRV) algorithms, QRS complexes were detected on the ECG traces and the apex of R waves were located using parabolic interpolation; this allowed the computation of heart period on a beat-to-beat basis. We carefully checked the ECG traces in order to avoid ectopic beats, artefacts or missing beats. Respirogram was obtained by resampling thorax and abdominal movements in correspondence of each R peak. Only ECG segments associated with stable and regular breathing were taken into account for the autonomic analysis. Stationary segments of 250±50 beats were selected for each patient and analysed using two different algorithms, linear spectral and non-linear symbolic analysis respectively. As to stationarity, this has been tested automatically for each segment by the program before analysis.

**Assessment of cardiac autonomic control**

HRV has been widely applied in both physiological and pathological conditions to evaluate autonomic cardiac control.^3^ Cardiac autonomic control was evaluated using a classical linear spectral analysis and a more recent non-linear symbolic analysis.

*Spectral analysis*

Total variability is the sum of the oscillatory components embedded in the heart period time series. Spectral analysis of HRV assesses the rhythmic oscillatory components that characterize the heart period time series. By means of an autoregressive model, we recognize three main oscillations embedded in heart period time series: very low frequency component (VLF) (frequency band below 0.04 Hz), low frequency component (LF) (frequency band between 0.04-0.15 Hz), and high frequency component (HF), synchronous with respiration. It has been clearly established that LF is a marker of sympathetic modulation while HF a marker of vagal modulation synchronous with respiration.^3^ For each oscillation, we can described a specific frequency and amplitude, which can be expressed in absolute units (ms^2^) and in normalized units (nu), with normalized units representing the relative values of each spectral component (i.e. LF and HF) with respect to the total power. Normalized units are calculated as follow: LF nu= [LF absolute units / (total power – VLF power)] and the HF nu = [HF absolute units / (total power-VLF)]. The ratio between LF and HF power can be calculated and it is considered marker of the sympatho-vagal balance.^3^

*Symbolic analysis*

Symbolic analysis is a non-linear method able to evaluate autonomic cardiac modulation and to detect non-reciprocal changes of sympathetic and parasympathetic modulations on heart period time series.^4^ The mathematical details have been described elsewhere.^4^ Symbolic analysis is based on the transformation of time series into a sequence of symbols, the construction of patterns (i.e. words), the reduction of the number of patterns into four families and the evaluation of their rate of occurrence. All patterns are grouped without any loss into four families: 0V, pattern with no variation (i.e. all the symbols are equal or in the same level), 1V, pattern with one variation (two consecutive symbols are equal and the remaining one is different), 2LV, patterns with two like variations (the three symbols form an ascending or descending ramp) and 2UV, patterns with two unlike variations (the three symbols form a peak or a valley). Their rate of occurrence is expressed as a percentage (i.e. 0V%, 1V%, 2LV%, and 2UV%). Experimental studies have demonstrated that 0V% is a marker of sympathetic modulation while 2LV% and 2ULV% are markers of vagal modulation.^4^

**Study definitions**

*Community-acquired pneumonia* (CAP) was defined as the presence of new pulmonary infiltrate seen on radiograph or computed tomography scan of the chest within 48 hours after hospitalization *plus* at least one of the following: a) new or increased cough with/without sputum production; b) fever (documented temperature -rectal or oral- ≥ 38.3 °C) or hypothermia (documented temperature –rectal or oral- <36 °C); c) evidence of systemic inflammation (such as abnormal white blood cell count -either leukocytosis (>10,000/cm3) or leukopenia (< 4,000/cm^3^) - or C-reactive protein or procalcitonin values above the local upper limit. *Severe sepsis* was defined by the presence of at least one of the following signs of organ hypoperfusion or organ dysfunction: 1) sepsis-induced hypotension; 2) lactate greater than the upper limits of normal laboratory results; 3) urine output <0.5 mL/kg/hour for >2 hours, despite adequate fluid resuscitation; 4) creatinine >2.0 mg/dL; 5) bilirubin >2 mg/dL; 6) platelet count <100,000; 7) coagulopathy (INR >1.5).^5^ *Acute respiratory failure* was defined as the presence of at least one among the following on admission: 1) partial pressure of oxygen in arterial blood (PaO_2_) < 60 mmHg; 2) ratio of PaO_2_ and fraction of inspired oxygen (PaO_2_/FiO_2_) < 250; 3) oxygen saturation < 90%; 4) respiratory acidosis. Respiratory acidosis was considered when a pH value on admission of less than 7.35 was identified with a partial pressure of carbon dioxide in arterial blood ≥ 45 mmHg.^6^ *Severe CAP* was defined according to 2007 American Thoracic Society/Infectious Diseases Society of America guidelines.^7^

**Definitions of cardiovascular events**

Cardiovascular events (CVE) were considered if any of the following was present either on hospital admission or during hospitalization: 1) acute myocardial infarction (AMI); 2) acute cardiogenic pulmonary edema (ACPE); 3) new arrhythmia; 4) acute worsening of a long-term arrhythmia.

Criteria for *AMI* were defined as: detection of rise and/or fall of troponin with at least one value above the 99th percentile of the upper reference limit together with evidence of myocardial ischemia with at least one of the following: 1) symptoms of ischemia; 2) ECG changes indicative of new ischemia (new ST-T changes or new left bundle branch block); 3) development of pathologic Q waves in the ECG; or 4) imaging evidence of new loss of viable myocardium or new regional wall motion abnormality.^8^

Diagnosis of *acute cardiogenic pulmonary edema* was established on the basis of medical history (acute severe dyspnea) and typical physical findings (widespread pulmonary rales), with chest radiography, performed within 6 hours from the event, confirming pulmonary vascular congestion.

Diagnosis of a *new arrhythmia* was established if any of the following was present as new onset: atrial flutter, atrial fibrillation, junctional supraventricular, or ventricular tachycardia.

Diagnosis of *an acute worsening of a long-term arrhythmia* was established if atrial fibrillation was switched to atrial flutter or in the presence of a switch of classes in Lown classification or in the presence of hemodynamic impairment (clinical features of shock and/or systolic blood pressure< 90 mmHg).

All of the enrolled patients were evaluated on admission and every day during hospitalization to identify those undergoing CVE. If a patient was diagnosed on admission to the hospital as having both CAP and a CVE, the latter was defined to be on hospital admission. Each case of cardiovascular event was presented and discussed to a clinical review committee in each center, composed of at least three physicians, one of whom was a cardiologist. Electrocardiogram and Troponin T or I were performed for the enrolled patients on hospital admission, and at every moment when clinicians had a clinical suspicion of the presence of a cardiovascular event. Troponin T or I determinations were performed with 3 set (every 6 hours) during the first 24 hours after hospital admission and if a suspicion of AMI was present during the hospital course. Chest radiograph was performed during hospital course if a suspicion of ACPE was present.

**Study outcome**

A patient was considered to reach clinical stability (CS) when the following criteria were met in a single day during hospitalization: 1) improved clinical signs (cough and shortness of breath); 2) patient will be afebrile for at least eight hours; 3) improving leukocytosis (decreased at least 10% from the previous day) or C-reactive protein or procalcitonin; 4) tolerating oral intake. Criteria for CS were evaluated daily during the first seven days of hospitalization.

**Statistical analysis**

Univariate and multivariate logistic regression analyses were performed to assess association between the time to clinical stability (dichotomized, ≤7days vs >7 days) and the following variables: heart rate (HR), total power (TP),VLF, LFa, HFa, LFnu, HFnu, LF/HF, central frequency of respiration (RESPHF), 0V%, 1V%, 2LV%, 2UV% and Pneumonia Severity Index. Only the variables significantly associated with the time to clinical stability in univariate analysis were considered in the multivariate analysis. A backward elimination procedure was used to find the best multivariate model. Odds ratios (OR) for the risk of reaching clinical stability after 7 days, with their 95% confidence intervals (CI), were calculated. The overall performance of the multivariate logistic models (ability of the model to discriminate between patients with TCS>7 and patients with TCS≤7 days) was assessed calculating the c statistic, ranging from 0.5 (chance discrimination) to 1.0 (perfect discrimination). Similar analyses were performed to assess the association between the presence of severe CAP on hospital admission and the above reported variables. ORs (with 95% CI) for the risk of having severe CAP were calculated.

*Sample size calculation*

Enrolling 75 patients, considering TCS>7 as the event of interest in our analysis, and assuming 30% of patients with TCS>7 days, we have planned to observe 22 patients with TCS>7. Having more than 20 patients with the event of interest, we have planned to perform a multivariate logistic regression analysis with the inclusion of at least two covariates.^9^

**REFERENCES**

1 Fine MJ, Auble TE, Yealy DM, et al. A prediction rule to identify low-risk patients with community-acquired pneumonia. *N Engl J Med.* 1997; 336: 243-250.

2 Voss A, Schroeder R, Heitmann A, Peters A, Perz S. Short-term heart rate variability--influence of gender and age in healthy subjects. *PLoS One.* 2015; 10: e0118308.

3 Montano N, Porta A, Cogliati C, et al. Heart rate variability explored in the frequency domain: a tool to investigate the link between heart and behavior. *Neurosci Biobehav Rev.* 2009; 33: 71-80.

4 Porta A, Tobaldini E, Guzzetti S, Furlan R, Montano N, Gnecchi-Ruscone T. Assessment of cardiac autonomic modulation during graded head-up tilt by symbolic analysis of heart rate variability. *Am J Physiol Heart Circ Physiol.* 2007; 293: 702-708.

5 Dellinger RP, Levy MM, Carlet JM, et al. Surviving Sepsis Campaign: international guidelines for management of severe sepsis and septic shock: 2008. *Crit Care Med.* 2008; 36: 296-327.

6 Aliberti S, Brambilla AM, Chalmers JD, et al. Phenotyping community-acquired pneumonia according to the presence of acute respiratory failure and severe sepsis. *Respir Res.* 2014; 15: 27.

7 Mandell LA, Wunderink RG, Anzueto A, et al. Infectious Diseases Society of America/American Thoracic Society consensus guidelines on the management of community-acquired pneumonia in adults. *Clin Infect Dis.* 2007; 44 Suppl 2: S27-72.

8 Thygesen K, Alpert JS, White HD, et al. Universal definition of myocardial infarction. *Circulation.* 2007; 116: 2634-2653.

9 Altman DG. Practical statistics for medical research. London: Chapman & Hall; 1991.

**Table S1. Autonomic cardiac parameters in patients with and without severe community-acquired pneumonia (CAP) on hospital admission.**

|  | **Non severe CAP**  **n=56** | **Severe CAP**  **n=19** | **p** |
| --- | --- | --- | --- |
| **Parameters** |  |  |  |
| Heart rate, bpm | 78 (68-88) | 83 (80-98) | 0.02 |
| **Spectral analysis** |  |  |  |
| Total power, ms^2^ | 149 (83-358) | 182 (72-408) | 0.70 |
| VLF, ms^2^ | 84 (9-177) | 13 (0-41) | 0.004 |
| LFnu | 40 (14-64) | 17 (2-63) | 0.07 |
| HFnu | 43 (18-59) | 37 (11-63) | 0.60 |
| LF/HF | 0.9 (0.2-2.5) | 0.7 (0.04-3.9) | 0.30 |
| RESPHF | 0.31 (0.27-0.34) | 0.35 (0.28-0.49) | 0.20 |
| **Symbolic analysis** |  |  |  |
| 0V% | 28 (16-39) | 16 (6-35) | 0.04 |
| 1V% | 42(34-48) | 37 (31-41) | 0.04 |
| 2LV% | 4 (2-7) | 6 (3-13) | 0.10 |
| 2UV% | 21 (12-34) | 38 (19-47) | 0.01 |

All the data are presented as median (IQR). VLF: very low frequency; LF: low frequency; HF: high frequency.

**Table S2. Univariate logistic regression analysis for the presence of severe CAP on hospital admission.**

|  | **OR (95% CI)** | **p value** |
| --- | --- | --- |
| Heart rate, bpm | 1.039 (1.003 – 1.077) | 0.035 |
| Total power, ms^2^ | 1.000 (0.998 – 1.001) | 0.659 |
| VLF, ms^2^ | 0.989 (0.980 – 0.999) | 0.031 |
| LFa, ms^2^ | 1.002 (0.998 – 1.005) | 0.349 |
| HFa, ms^2^ | 0.999 (0.995 – 1.003) | 0.727 |
| *LFnu* | 0.985 (0.966 – 1.004) | 0.129 |
| *HFnu* | 0.996 (0.977 – 1.015) | 0.670 |
| *LF/HF* | 1.017 (0.945 – 1.095) | 0.651 |
| *RESPHF* | 1.063 (1.007 – 1.122) | 0.027 |
| *0V%* | 0.973 (0.942 – 1.005) | 0.093 |
| 1V% | 0.956 (0.907 – 1.007) | 0.092 |
| *2LV%* | 1·073 (0.979 – 1.176) | 0.132 |
| *2UV%* | 1·041 (1.007 – 1.076) | 0.017 |

VLF: very low frequency; LFa: low frequency absolute units; LFnu: low frequency normalized units; HFa: high frequency absolute units; HFnu: high frequency normalized units; RESPHF: central frequency of respiration.

**Table S3. Univariate logistic regression analysis for the risk of time to clinical stability > 7 days.**

|  | OR (95% CI) | p value |
| --- | --- | --- |
| Heart rate, bpm | 1.017 (0.986 – 1.049) | 0.289 |
| Total power, ms^2^ | 0.997 (0.994 – 1.000) | 0.039 |
| VLF, ms^2^ | 0.989 (0.980 – 0.997) | 0.009 |
| LFa, ms^2^ | 0.995 (0.989 – 1.001) | 0.107 |
| HFa, ms^2^ | 0.996 (0.990 – 1.002) | 0.178 |
| LFnu | 0.999 (0.982 – 1.015) | 0.869 |
| HFnu | 1.003 (0.985 – 1.020) | 0.764 |
| LF/HF | 0.966 (0.889 – 1.050) | 0.419 |
| RESPHF | 1.096 (1.031 – 1.166) | 0.004 |
| 0V% | 0.966 (0.937 – 0.995) | 0.023 |
| 1V% | 0.994 (0.948 – 1.041) | 0.789 |
| 2LV% | 1.077 (0.987 – 1.175) | 0.098 |
| 2UV% | 1.035 (1.004 – 1.066) | 0.028 |
| PSI | 1.010 (0.998 – 1.022) | 0.089 |

VLF: very low frequency; LFa: low frequency absolute units; LFnu: low frequency normalized units; HFa: high frequency absolute units; HFnu: high frequency normalized units; RESPHF: central frequency of respiration; PSI: pneumonia severity index.
